# ADVANCE DIRECTIVES: SOLO AGERS RISK OF BECOMING "UNBEFRIENDED"

**DOI:** 10.1093/geroni/igac059.2010

**Published:** 2022-12-20

**Authors:** Rupal Parekh, Kathryn Betts Adams, Donna Schuman

**Affiliations:** University of Connecticut, West Hartford, Connecticut, United States; University of Connecticut, West Hartford, Connecticut, United States; University of Texas at Arlington, Arlington, Texas, United States

## Abstract

Solo agers are an at-risk population of almost 14 million older U.S. adults currently living alone (Administration for Community Living, 2018). Having been independent for most of their lives they often enter older age unprepared for declining health. Even though the numbers of solo agers are rapidly increasing with the aging of the Baby Boomer generation, research remains scant on this population (Colby & Ortman, 2014). A mounting concern facing solo agers is finding themselves “unbefriended” (i.e., having no one to act as health care proxy in the event of incapacitation due to a medical crisis). The risk of unbefriended status emphasizes the critical importance of advance care planning; however, many solo agers have no advance directives and factors influencing advance care planning in this group are unclear. In this mixed-methods study, we examined factors influencing advance care planning among solo agers. Survey data were collected from 467 members of a Facebook group for self-identified “elder orphans,” six of whom subsequently participated in qualitative interviews. Among these solo agers, 55% indicated they had advance directives. Hierarchical logistic regression results indicated financial and overall wellbeing predicted having advance directives; however, perceived health risk did not. An interpretive phenomenological analysis of data from six in-depth interviews revealed emergent themes of fears of the future and reluctance to plan for end of life despite acknowledging health risks. Findings can inform policies to meet the growing needs of solo agers who may be at elevated risk of becoming unbefriended.

